# Safety Assessment of *Pseudomonas fluorescens* DS17R Chloroform Extract: Low Acute But Dose‐Dependent Subacute Oral Toxicity in Rats

**DOI:** 10.1155/jt/8893441

**Published:** 2026-04-23

**Authors:** Samuel Arsène Ntyam Mendo, Séverin Tchameni Nguemezi, Laure Brigitte Kouitcheu Mabeku, Modeste Lambert Sameza, Rosalie Anne Ngono Ngane

**Affiliations:** ^1^ Department of Biological Science, Higher Teacher Training College, University of Yaounde 1, P.O. Box 47, Yaounde, Cameroon, uy1.uninet.cm; ^2^ Department of Biochemistry, Faculty of Sciences, University of Douala, P.O. Box 24 157, Douala, Cameroon, univ-douala.cm; ^3^ Department of Microbiology, Faculty of Science, University of Yaounde 1, P.O. Box 812, Yaounde, Cameroon, uy1.uninet.cm

**Keywords:** biocontrol agent, microbial extract, OECD guidelines, *Pseudomonas fluorescens*, subacute toxicity

## Abstract

The present study evaluated the acute and subacute oral toxicities of a chloroform extract from *Pseudomonas fluorescens* DS17R in Wistar rats to support its safe application as a biocontrol agent. For acute toxicity assessment following OECD Guideline 423, female Wistar rats (*n* = 3 per step) received single oral doses of 300, 2000, or 5000 mg/kg body weight (bw) and were observed for 14 days. For subacute toxicity, female rats (*n* = 5 per group) received daily oral doses of 0 (control), 57.5, 115, 230, or 460 mg/kg bw for 28 days, after which hematological, biochemical, and histopathological analyses were performed. Acute toxicity testing revealed no mortality at 300 or 2000 mg/kg bw, but 50% mortality at 5000 mg/kg bw, yielding a median lethal dose (LD_50_) of 4574 mg/kg bw, classifying the extract as practically nontoxic (OECD Category 5). Subacute 28‐day oral exposure induced dose‐ and time‐dependent physiological and biochemical responses. Body weight showed a biphasic pattern, with a significant increase at 57.5 mg/kg bw and reductions at ≥ 115 mg/kg bw, while feed intake was transiently suppressed. Hepatic enlargement was observed at 115–460 mg/kg bw, accompanied by elevated alkaline phosphatase and mild AST increase; histology revealed sinusoidal dilation and leukocyte infiltration. Splenomegaly, lymphopenia at 230 and 460 mg/kg bw, microcytosis, anisocytosis, and thrombocytosis reflected hematopoietic and immune modulation. Renal stress was evidenced by hyponatremia, hypokalemia, hypochloremia, hypercalcemia, and decreased phosphate at doses ≥ 57.5 mg/kg bw, with mild tubular alterations. Lipid remodeling included increased triglycerides and HDL, with decreased LDL at doses ≥ 115 mg/kg bw. Overall, the extract induced adaptive metabolic and redox‐mediated responses without irreversible organ damage, supporting its potential safe use as a microbial biocontrol agent under the tested conditions.

## 1. Introduction

The intensification of modern agriculture has been accompanied by an extensive reliance on synthetic pesticides, which have raised serious concerns due to their persistent environmental contamination, disruption of ecological equilibrium, and adverse impacts on human and animal health [1, 2]. These concerns have catalyzed a global search for sustainable and ecologically sound pest management strategies, aimed at reducing dependence on hazardous agrochemicals while supporting resilient and productive agroecosystems [3, 4]. Among the most promising alternatives, biological control, which exploits the natural antagonistic capacities of beneficial microorganisms to suppress plant pathogens and pests, has emerged as a cornerstone of integrated pest management and sustainable agriculture [3, 5]. Within this paradigm, plant growth‐promoting rhizobacteria (PGPR), especially fluorescent pseudomonads, have attracted substantial scientific and practical interest because of their broad‐spectrum antagonistic activities against a wide range of phytopathogens [6–8]. Recent genomic and metabolomic analyses have further elucidated the strain‐level diversity and secondary metabolite repertoires of biocontrol pseudomonads, revealing complex and context‐dependent antagonistic mechanisms [9]. In disease‐suppressive soils which inherently inhibit the establishment and progress of soilborne pathogens members of the genus *Pseudomonas* are frequently identified as dominant biocontrol agents [6, 10]. The efficacy of these bacteria is attributed to a diverse repertoire of bioactive secondary metabolites, including well‐characterized antibiotics such as 2,4‐diacetylphloroglucinol (2,4‐DAPG), phenazine‐1‐carboxylic acid (PCA), pyrrolnitrin, and pyoluteorin, as well as siderophores, biosurfactants, and hydrogen cyanide (HCN) [11–13]. These metabolites act through multiple modes of antagonism, ranging from direct inhibition of pathogen growth to induction of host systemic resistance, thereby enhancing both the protective and competitive fitness of *Pseudomonas* spp. in the rhizosphere [7, 12]. Despite their ecological and agronomic potential, the very biochemical complexity that renders fluorescent *Pseudomonas* spp. effective biocontrol agents also raise important toxicological questions. Although the antagonistic properties of these bacteria have been widely documented in plant pathology and microbiology literature, systematic evaluations of the mammalian safety of nonpathogenic strains intended for agricultural application remain limited [14–16]. The bulk of existing toxicological knowledge derives from studies of *Pseudomonas aeruginosa*, a recognized human opportunistic pathogen that produces virulence determinants including pyocyanin, lipopolysaccharide (LPS), and rhamnolipids, which are capable of inducing immune dysregulation, cellular injury, and systemic inflammatory responses in mammalian hosts [17–19]. By contrast, the potential of certain *P. fluorescens* strains to produce metabolites with analogous bioactivities such as HCN and extracellular enzymes with poorly understood systemic effects necessitates careful safety evaluation rather than the assumption of benignity [14, 20]. Recent mechanistic investigations have demonstrated that phenazine‐type metabolites, characteristic of fluorescent pseudomonads, can undergo redox cycling to generate reactive oxygen species, leading to oxidative stress, mitochondrial dysfunction, and immunomodulatory effects in mammalian systems [21]. The regulatory landscape further underscores the need for strain‐specific toxicological data. Although select *P. fluorescens*–based products are registered and commercialized as biopesticides or biofertilizers in some jurisdictions, regulatory acceptance under frameworks in the European Union remains inconsistent, in part due to gaps in rigorous, guideline‐compliant safety datasets [22–24]. Notably, detailed acute and subacute toxicity studies conducted according to internationally recognized protocols such as those established by the Organisation for Economic Co‐operation and Development (OECD) are scarce for *P. fluorescens* extracts or metabolites [25, 26]. This paucity of data presents a critical barrier to robust risk assessment and regulatory approval. In preceding work, we isolated and biochemically characterized fluorescent *Pseudomonas* strains with potent antagonistic activity against *Phytophthora colocasiae*, including a particularly efficacious isolate designated DS17R [27]. Chloroform extracts obtained from *P. fluorescens* DS17R exhibited strong antimicrobial activity and notable thermostability [27], and phytochemical analyses revealed a complex profile of bioactive compounds including phenazines, flavonoids, and phenolic constituents likely underpinning its antagonistic efficacy [28]. While this chemical diversity affirms the strain’s potential as a biocontrol agent, it simultaneously highlights the imperative for rigorous evaluation of mammalian toxicological effects, especially under conditions of acute or repeated oral exposure. Accordingly, the present study was designed to address this critical knowledge gap by performing a comprehensive assessment of the acute and subacute oral toxicities of the characterized chloroform extract of Pseudomonas fluorescens DS17R in Wistar rats, conducted in strict accordance with OECD guideline protocols. The findings aim to generate essential toxicological data to inform risk assessment and to support the safe and responsible integration of this promising biocontrol agent into sustainable agricultural practice.

## 2. Materials and Methods

### 2.1. Bacterial Strain and Extract Preparation

The fluorescent *Pseudomonas fluorescens* strain DS17R, previously isolated and characterized for its biocontrol activity against *Phytophthora colocasiae* [28, 29], was used in this study. The strain was cultured in King’s B broth (HiMedia, India) at 28°C for 48 h under constant agitation (150 rpm). Secondary metabolites were extracted from the bacterial supernatant using chloroform, following the method described by Olanbiwoninu et al. [30] with minor modifications. Briefly, the supernatant was filter‐sterilized (0.22 μm pore size) and mixed with an equal volume of chloroform (1:1 v/v). The mixture was shaken vigorously for 2 h, after which the organic phase was collected. The chloroform was evaporated under reduced pressure at 40°C, and the resulting crude extract was lyophilized. The extract was stored at −20°C until use. This specific extract has been previously phytochemically characterized, confirming the presence of phenazines, flavonoids, and phenolic compounds [29].

### 2.2. Animal Models

Healthy, nulliparous, and nonpregnant female Wistar rats (8–10 weeks old, 115–140 g) were obtained from a certified breeding facility (ISO 9001:2015 certified). Animals were housed in sterile polypropylene cages under strictly controlled environmental conditions: temperature of 22 ± 2°C, relative humidity of 55 ± 10%, and a 12 h light/dark cycle. They were provided with ad libitum access to a standard rodent diet (AIN‐93M) and filtered water. The study protocol was approved by the Institutional Animal Ethics Committee, and all procedures were conducted in strict compliance with the OECD Guidelines for the Testing of Chemicals [31, 32] and the ARRIVE 2.0 guidelines [33]. Female rats were selected for the subacute study due to their recognized higher sensitivity to toxicants, as recommended by OECD Guideline 407 [31].

### 2.3. Acute Toxicity Study

#### 2.3.1. Experimental Design

Acute oral toxicity was assessed according to OECD Guideline 423 [7] (Acute Oral Toxicity‐Acute Toxic Class Method). Female Wistar rats (nulliparous, nonpregnant, 8–10 weeks old) were used exclusively, as recommended by the guideline due to their recognized higher sensitivity to toxicants. Animals were fasted overnight (12–16 h) prior to dosing, with free access to water. The *P. fluorescens* DS17R chloroform extract was suspended in distilled water and administered via oral gavage at a constant volume of 10 mL/kg body weight.

The study followed a sequential dosing procedure: Step 1. Three female rats received 300 mg/kg bw and were observed for 48 h. No mortality or severe toxicity was observed. Step 2. A second group of three female rats received 2000 mg/kg bw. Again, no mortality occurred within 48 h. Step 3. A third group of three female rats received 5000 mg/kg bw (the limit dose). Mortality was observed within 72 h.

All animals were observed individually for mortality and clinical signs (changes in skin, fur, eyes, mucous membranes, respiratory rate, and autonomic and central nervous system function) at 0.5, 1, 2, 4, 6, 12, and 24 h postdosing, and daily thereafter for 14 days. Body weight was recorded weekly. At the end of the observation period, surviving animals were euthanized by cervical dislocation under mild chloroform anesthesia. According to the OECD 423 classification system, when mortality occurs at 5000 mg/kg, the test substance is classified as Category 5 (LD50 > 2000 but ≤ 5000 mg/kg). The median lethal dose (LD_50_) was estimated using probit analysis [34] to provide a quantitative reference point.

#### 2.3.2. Observations and LD_50_ Determination

Animals were observed meticulously for mortality, clinical signs of toxicity (lethargy, piloerection, respiratory distress, ataxia, and tremors), and behavioral changes at 0.5, 1, 2, 4, and 24 h postadministration, and then daily for a total of 14 days. Body weight, food, and water intake were recorded daily. Survivors were euthanized on Day 14 by cervical dislocation under mild chloroform anesthesia, adhering to established humane endpoints. The median LD_50_ was calculated using the probit regression method of Miller and Tainter [34].

### 2.4. Subacute Toxicity Study

#### 2.4.1. Experimental Design

Doses for the 28‐day repeated‐dose oral toxicity study were selected based on established toxicological principles and the results of the acute toxicity study, following OECD Guideline 407 recommendations. Accordingly, in the absence of prior subchronic toxicity data, the highest dose was set at approximately one‐tenth of the acute LD_50_ (4574 mg/kg bw), a standard conservative fraction in regulatory toxicology when transitioning from acute to repeated‐dose studies, as this level is expected to produce observable toxic effects without causing excessive mortality or suffering; the value was adjusted from 457.4 mg/kg to 460 mg/kg bw for practical dosing convenience. Three additional lower doses were then selected using a geometric progression with a common ratio of two: 230 mg/kg bw (1/20 of the LD_50_), 115 mg/kg bw (1/40 of the LD_50_), and 57.5 mg/kg bw (1/80 of the LD_50_). This logarithmic spacing is standard in toxicology to ensure adequate coverage of the dose‐response curve and to identify both the No‐Observed‐Adverse‐Effect Level (NOAEL) and the Lowest‐Observed‐Adverse‐Effect Level (LOAEL). The resulting dose range (57.5 to 460 mg/kg bw) encompasses approximately two orders of magnitude below the acute LD_50_, allowing detection of effects that may manifest only after repeated exposure at doses well below the acute lethal threshold. It is important to note that this study evaluates a crude bacterial extract intended for agricultural application as a biocontrol agent; unlike pharmaceutical development, there is no established “therapeutic dose” in humans, and therefore dose selection was based exclusively on toxicological principles to characterize the hazard profile of the substance rather than to establish a therapeutic window. This dose selection approach (1/10 to 1/80 of the acute LD_50_) is consistent with published guidelines for toxicity testing of botanical and microbial extracts where no prior human exposure data exist [35].

#### 2.4.2. Physiological Monitoring

Body weight and weekly feed and water consumption were measured throughout the study. The percentage weight gain was calculated as ([final weight − initial weight]/Initial weight) × 100.

#### 2.4.3. Organ Collection and Weighing

On Day 28, after a 24 h fasting period, animals were then anesthetized with ether and sacrificed. Rats were then placed in a supine position, the abdominal cavity opened, the intestines moved to the left, the abdominal aorta was located, a 10 mL syringe needle was inserted at the base of the aorta, and immediately the maximum amount of blood was collected, and the animal’s death was verified [36]. Blood was collected via cardiac puncture into two tubes: EDTA‐coated tubes for hematological analysis and plain tubes for serum separation. Serum was obtained by centrifugation at 2500 × g for 15 min at 4°C. Major organs (liver, kidneys, spleen, heart, and lungs) were excised, rinsed in ice‐cold phosphate buffer (0.1 M, pH 7.4), blotted dry, and weighed. Relative organ weights were calculated as (absolute organ weight/final body weight) × 100. Sections of the liver and kidneys were fixed in 10% neutral‐buffered formalin for histopathological examination.

#### 2.4.4. Hematological and Biochemical Assays

Hematological parameters, including red blood cell (RBC) count, white blood cell (WBC) count, red cell distribution width (RDW), hemoglobin (HGB), packed cell volume (PCV), platelet count (PLT), and red cell indices (MCV, MCH, and MCHC), were analyzed using an automated hematology analyzer (Sysmex XS‐1000i, Japan). Serum biochemical parameters alanine aminotransferase (ALT), aspartate aminotransferase (AST), alkaline phosphatase (ALP), creatinine, urea, total protein, albumin, bilirubin, glucose, total cholesterol, triglycerides, high‐density lipoprotein (HDL) cholesterol, and electrolytes (sodium, potassium, chloride, calcium, and phosphate) were measured using commercial diagnostic kits (BIOLABO SAS and LABCARE Diagnostics) on a clinical chemistry analyzer.

#### 2.4.5. Histopathological Examination

Formalin‐fixed tissues were processed by standard histological techniques: dehydrated in a graded ethanol series, cleared in xylene, and embedded in paraffin. Tissue sections (5 μm thick) were stained with Hematoxylin and Eosin (H&E). Slides were examined under a light microscope (Leica DM500) by a pathologist blinded to the treatment groups. Lesions were scored and described according to established criteria.

### 2.5. Statistical Analysis

All data are presented as mean ± standard error of the mean (SEM). Statistical analyses were performed using GraphPad Prism software (Version 9.0) and R Version 4.5.2 . Differences between the treatment groups and the control were assessed by one‐way analysis of variance (ANOVA) followed by Tukey’s post hoc test for multiple comparisons. A value of *p* < 0.05 was considered statistically significant.

## 3. Results

### 3.1. Acute Toxicity Assessment

Acute oral toxicity of the *Pseudomonas fluorescens* DS17R chloroform extract was evaluated in female Wistar rats following the sequential dosing procedure of OECD Guideline 423. Administration of the extract at 300 mg/kg bw resulted in no mortality and only transient, mild clinical signs (hypoactivity in 1/3 animals, resolved within 24 h). At 2000 mg/kg bw, no mortality was observed, although dose‐dependent clinical signs including hypoactivity (3/3 animals), piloerection (2/3), and shallow respiration (1/3) were noted, all resolving within 48 h. In contrast, the 5000 mg/kg bw dose group exhibited severe toxicity, characterized by persistent hypoactivity (3/3 animals), ataxia (3/3), and tremors (2/3), culminating in 66.7% mortality (2 of 3 animals) within 72 h. No further mortality occurred during the remaining 14‐day observation period. Surviving animals showed gradual recovery of clinical signs by Day 7. Based on these results, the extract is classified as OECD Category 5 (LD50 > 2000 but ≤ 5000 mg/kg), corresponding to the statement practically nontoxic in the Globally Harmonized System (GHS) of classification. The median LD_50_ was estimated at 4574 mg/kg bw using probit analysis (95% confidence interval: 3892–5102 mg/kg bw; *R* ^2^ = 0.98), providing a quantitative reference for dose selection in the subacute study.

### 3.2. Subacute Toxicity Findings

#### 3.2.1. Body Weight Evolution and Feed Intake

##### 3.2.1.1. Body Weight

**FIGURE 1:**
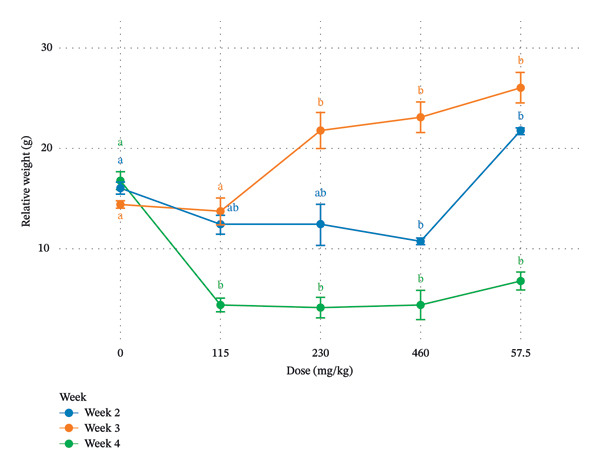
Dose‐ and time‐dependent effects of *Pseudomonas fluorescens* DS17R chloroform extract on body weight evolution in female Wistar rats. Data represent mean relative body weight (g) ± SEM (*n* = 5 per group) measured at Weeks 2, 3, and 4 of the 28‐day oral administration period. Relative body weight was calculated as the percentage change from initial body weight at study commencement. Statistical analysis was performed using two‐way ANOVA (factors: dose and time) followed by Tukey’s HSD post hoc test for multiple comparisons. Within each time point (week), bars bearing different letters (a, b) indicate statistically significant differences between dose groups (*p* < 0.05). Groups sharing the same letter are not significantly different. The control group (0 mg/kg bw) received vehicle only (distilled water, 10 mL/kg bw). A biphasic response is evident: at Week 2, a significant increase in body weight was observed at 57.5 mg/kg compared to intermediate doses; by Week 4, significant reductions were observed at doses ≥ 115 mg/kg bw, with the most pronounced decrease at 115 mg/kg bw, suggesting cumulative or delayed effects.

Figure 1 illustrates the evolution of relative body weight (g) as a function of administered dose (mg/kg) over three consecutive weeks (Week 2, Week 3, and Week 4). At 0 mg/kg bw, relative weights were comparable across the three time points, with no marked statistical divergence, indicating homogeneity prior to treatment.

At Week 2, relative weight showed a moderate decline at 115 mg/kg bw and remained relatively stable at 230 mg/kg bw, followed by a slight decrease at 460 mg/kg. Interestingly, at 57.5 mg/kg bw, a pronounced increase in body weight was observed compared with intermediate doses, suggesting a possible biphasic or hormetic response. Statistical difference observed indicates partial overlap between certain dose levels but significant reduction at 460 mg/kg bw. At Week 3, a clear dose‐dependent increase in relative weight was observed from 115 to 57.5 mg/kg bw, with the highest value recorded at 57.5 mg/kg bw. Doses ≥ 230 mg/kg bw formed a statistically distinct group, significantly higher than the lower‐dose groups(115 and 57.5 mg/kg bw). In contrast, Week 4 demonstrated a marked reduction in relative weight at 115, 230, and 460 mg/kg bw compared to control. These groups were statistically homogeneous but significantly lower than baseline values. The reduction was particularly pronounced at 115 mg/kg bw, indicating potential cumulative or delayed toxic effects. Although a slight recovery was noted at 57.5 mg/kg bw, values remained significantly lower than control.

##### 3.2.1.2. Feed Intake

**FIGURE 2:**
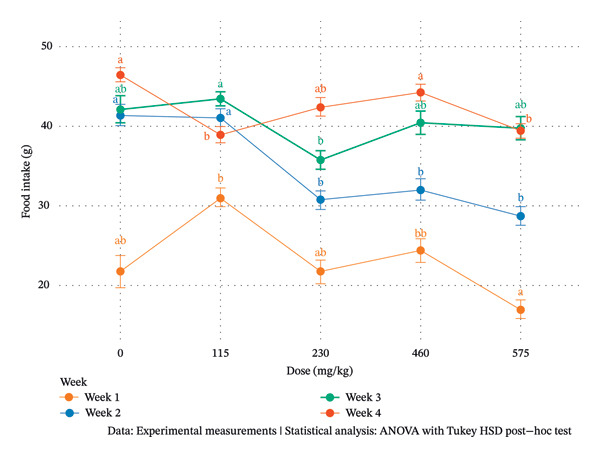
Temporal evolution of daily food intake in female Wistar rats during 28‐day oral administration of *Pseudomonas fluorescens* DS17R chloroform extract. Data represent mean daily food intake (g/rat/day) ± SEM (*n* = 5 per group) recorded weekly over the 4‐week treatment period. Statistical analysis was performed using two‐way ANOVA (factors: dose and week) followed by Tukey’s HSD post hoc test for multiple comparisons. Within each week, bars bearing different letters (a, b) indicate statistically significant differences between dose groups (*p* < 0.05). Groups sharing the same letter are not significantly different. The control group (0 mg/kg) received vehicle only (distilled water, 10 mL/kg bw). A time‐dependent pattern is evident: early exposure (Week 1) showed moderate fluctuations without clear dose‐response; Week 2 revealed significant suppression at 230 mg/kg bw; by Week 4, persistent reductions were observed at 57.5 and 115 mg/kg bw, while partial recovery occurred at 460 mg/kg bw, suggesting possible metabolic adaptation at the highest dose.

Figure 2 presents the evolution of food intake (g) across increasing doses (mg/kg) over four experimental weeks (Week 1 to Week 4). At Week 1, food intake showed moderate variability across doses without a clear monotonic dose‐response pattern. Intake increased at 115 mg/kg bw compared with control, declined at 230 mg/kg, slightly increased at 460 mg/kg bw, and reached the lowest value at 57.5 mg/kg bw. The statistical analysis indicates partial overlap among intermediate doses, suggesting limited early sensitivity to treatment during the first week.

At Week 2, a more pronounced dose‐related effect emerged. Food intake decreased markedly at 230 mg/kg bw and remained lower at 460 and 57.5 mg/kg bw compared with control and 115 mg/kg bw groups. The statistical homogeneity among higher doses suggests that exposure beyond 230 mg/kg bw induced a consistent reduction in feeding behavior. During Week 3, food intake exhibited a nonlinear pattern. A significant reduction was observed at 230 mg/kg bw, followed by partial recovery at 460 mg/kg bw and stabilization at 57.5 mg/kg bw. The persistence of lower intake at 230 mg/kg indicates sustained sensitivity at this dose level, whereas the rebound at 460 mg/kg bw may reflect adaptive metabolic compensation or behavioral adjustment.

By Week 4, a distinct temporal shift became evident. Food intake was highest in the control group and remained significantly elevated at 460 mg/kg bw, while a significant reduction was observed at 115 and 57.5 mg/kg bw. Interestingly, intake at 230 mg/kg bw remained intermediate, suggesting partial recovery compared with earlier weeks.

Overall, the data reveal a time‐dependent and nonmonotonic dose–response relationship. Early exposure induced moderate fluctuations, followed by a transient suppression of feeding at intermediate doses (notably 230 mg/kg bw), and later partial adaptation at higher doses.

#### 3.2.2. Organ Weights

Table 1 summarizes the dose‐dependent effects of *Pseudomonas fluorescens* DS17R chloroform extract on relative organ weights in female Wistar rats following 28 days of oral administration. No statistically significant differences were observed in heart and kidney relative weights across all tested doses compared with the control group. Although a slight reduction in heart weight was noted at 460 mg/kg, this variation remained nonsignificant. In contrast, the liver exhibited a clear and highly significant dose‐dependent increase in relative weight at all treated groups (*p* < 0.01 to *p* < 0.001). The progressive rise from 3.31  g (control) to 3.81  g at 230 mg/kg bw and 3.77  g at 460 mg/kg bw strongly suggests hepatomegaly. Lung relative weight increased significantly at 230 and 460 mg/kg bw (*p* < 0.05), indicating potential pulmonary involvement at higher exposure levels. Ovarian weights were significantly elevated at doses ≥ 115 mg/kg bw (*p* < 0.05), suggesting possible endocrine modulation or reproductive axis disturbance. Pancreatic weight showed a significant decrease at 57.5, 230, and 460 mg/kg bw (*p* < 0.05), whereas the 115 mg/kg bw group remained comparable to control.

**TABLE 1:** Dose‐response effects on relative organ weights in female Wistar rats following 28‐day oral administration of *P. fluorescens* DS17R chloroform extract.

Organs	Doses (mg/kg bw) of extract				
	Control	57.5	115	230	460
	Relative weight of organs (g)				
Heart	0.32 ± 0.01	0.346 ± 0.01^ns^	0.33 ± 0.01^ns^	0.34 ± 0.02^ns^	0.26 ± 0.02^ns^
Kidney	0.77 ± 0.01	0.75 ± 0.01^ns^	0.75 ± 0.01^ns^	0.77 ± 0.01^ns^	0.77 ± 0.04^ns^
Liver	3.31 ± 0.04	3.47 ± 0.01^∗∗^	3.58 ± 0.03^∗∗^	3.81 ± 0.06^∗∗^	3.77 ± 0.04^∗∗∗^
Lungs	0.703 ± 0.02	0.69 ± 0.01^ns^	0.76 ± 0.03^ns^	0.78 ± 0.02^∗^	0.80 ± 0.02^∗^
Ovaries	0.046 ± 0.004	0.046 ± 0.004^ns^	0.063 ± 0.003^∗^	0.069 ± 0.001^∗^	0.068 ± 0.002^∗^
Pancreas	0.62 ± 0.01	0.54 ± 0.01^∗^	0.60 ± 0.01^ns^	0.56 ± 0.01^∗^	0.57 ± 0.01^∗^
Spleen	0.296 ± 0.005	0.34 ± 0.01^∗^	0.35 ± 0.01^∗^	0.39 ± 0.01^∗^	0.52 ± 0.02^∗∗^
Stomach	0.463 ± 0.02	0.53 ± 0.01^∗^	0.45 ± 0.01^ns^	0.41 ± 0.02^ns^	0.45 ± 0.02^ns^

*Note:* Values represent mean ± SEM (*n* = 5). Statistical significance versus control: ^∗^ *p* < 0.05, ^∗∗^ *p* < 0.01, ^∗∗∗^ *p* < 0.001.

^ns^Not significant (one‐way ANOVA with Tukey’s post hoc test).

The spleen demonstrated a marked and dose‐dependent enlargement, reaching statistical significance at all treated doses and becoming highly significant at 460 mg/kg bw (*p* < 0.01). Finally, the stomach showed a significant increase only at 57.5 mg/kg bw (*p* < 0.05), with no significant differences at higher doses, suggesting a possible low‐dose localized adaptive or irritative response rather than progressive gastric toxicity.

#### 3.2.3. Hematological Parameters

Table 2 presents the dose‐dependent hematological alterations observed in female Wistar rats after 28 days of oral administration of *P. fluorescens* DS17R chloroform extract. Concerning erythrocyte‐related parameters, Hb levels showed a progressive and statistically significant increase from 115 mg/kg bw onward (*p* < 0.05 to *p* < 0.001), reaching a maximum at 460 mg/kg bw. This upward trend suggests enhanced Hb concentration per circulating volume. However, in contrast, red blood cell counts (RBCs) significantly decreased at doses ≥ 115 mg/kg bw, indicating a reduction in erythrocyte number despite increased Hb levels.

**TABLE 2:** Dose‐dependent alterations in hematological parameters of female Wistar rats following 28‐day oral administration of *P. fluorescens* DS17R chloroform extract.

Haematological parameters	Doses of extracts (mg/kg bw)				
	Control	57.5	115	230	460
HGB (g/L)	221.44 ± 4.52	219.57 ± 3.75^ns^	231.13 ± 3.99^∗^	242.00 ± 2.25^∗∗^	264.35 ± 6.31^∗∗∗^
RBC (× 10^12^/L)	880.01 ± 7.39	876.62 ± 4.45^ns^	861.66 ± 3.56^∗^	856.54 ± 6.66^∗^	844.24 ± 3.55^∗∗^
PCV (L/L)	1.45 ± 0.33	1.22 ± 0.04^∗^	1.20 ± 0.43^∗^	1.19 ± 0.65^∗^	1.12 ± 0.28^∗∗^
MGV (fL)	75.05 ± 1.88	68.35 ± 1.88^∗^	55.45 ± 3.33^∗∗^	52.62 ± 3.46^∗∗∗^	51.03 ± 0.50^∗∗∗^
MCH (pg)	21.00 ± 0.74	22.91 ± 3.41^ns^	19.01 ± 2.69^ns^	22.55 ± 0.20^ns^	17.63 ± 4.36^ns^
MCHC (g/L)	316.55 ± 6.92	319.00 ± 5.67^ns^	333.00 ± 4.78^∗^	351.66 ± 3.83^∗∗^	376.49 ± 2.45^∗∗∗^
RDW (%)	29.21 ± 1.66	41.72 ± 2.55^∗^	53.33 ± 2.20^∗∗^	55.01 ± 1.75^∗∗^	65.50 ± 1.88^∗∗∗^
WBC (× 10^3^/mm^3^)	80.00 ± 0.30	71.25 ± 1.79^∗^	68.66 ± 0.33^∗^	66.71 ± 0.56^∗^	61.31 ± 4.20^∗∗^
Neutro (× 10^9^/L)	11.43 ± 2.71	8.22 ± 0.44^ns^	8.00 ± 0.00^ns^	7.15 ± 0.29^ns^	7.05 ± 0.35^ns^
Lymph (× 10^9^/L)	42.00 ± 0.50	41.00 ± 0.00^ns^	39.66 ± 0.48^ns^	36.00 ± 1.52^∗^	33.25 ± 4.50^∗^
Monos (× 10^9^/L)	2.30 ± 0.35	3.31 ± 0.82^∗^	4.22 ± 0.33^∗∗^	4.19 ± 0.95^∗∗^	4.45 ± 0.67^∗∗∗^
Eosino(× 10^9^/L)	3.41 ± 0.66	3.4 ± 0.02^ns^	3.45 ± 0.05^ns^	3.43 ± 0.02^ns^	3.48 ± 0.03^ns^
PLT (× 10^9^/L)	736.55 ± 2.65	759.34 ± 7.75^∗^	789.10 ± 4.21^∗∗^	787.66 ± 3.55^∗∗^	793.23 ± 6.75^∗∗∗^

*Note:* Values represent mean ± SEM (*n* = 5). Statistical significance versus control: ^∗^ *p* < 0.05, ^∗∗^ *p* < 0.01, ^∗∗∗^ *p* < 0.001. PLT: platelets, HGB: hemoglobin, RDW: red cell distribution width.

Abbreviations: MCH, mean corpuscular hemoglobin; MCHC, mean corpuscular hemoglobin concentration; MCV, mean corpuscular volume; MPV, mean platelet volume; RBC, red blood cells; WBC, white blood cells.

^ns^Not significant (one‐way ANOVA with Tukey’s post hoc test).

Consistently, packed cell volume (PCV) significantly declined at all treated doses compared with control, demonstrating a reduction in total erythrocyte mass. Moreover, mean corpuscular volume (MCV) exhibited a marked and dose‐dependent decrease (*p* < 0.05 to *p* < 0.001), indicating microcytosis.

In parallel, while mean corpuscular hemoglobin (MCH) remained statistically unchanged, MCH concentration (MCHC) significantly increased in a dose‐dependent manner from 115 mg/kg bw upward. Taken together, the simultaneous reduction in MCV and increase in MCHC suggest the presence of microcytic but relatively hyperchromic erythrocytes.

Furthermore, red cell distribution width (RDW) increased markedly and dose‐dependently (*p* < 0.05 to *p* < 0.001), reflecting anisocytosis and heterogeneity in erythrocyte size. Turning to leukocyte parameters, total white blood cells count (WBC) significantly decreased across all treated groups. However, when differential counts are considered, neutrophils did not show statistically significant changes, indicating relative stability of this subpopulation.

In contrast, lymphocyte counts significantly decreased at higher doses (230 and 460 mg/kg bw), suggesting dose‐related lymphopenia. Conversely, monocyte counts increased significantly and dose‐dependently, reaching high statistical significance at 460 mg/kg bw. Notably, eosinophil levels remained stable across all groups, indicating that allergic or parasitic‐type immune responses were not prominently involved. Moreover, PLT exhibited a significant and progressive increase across doses (*p* < 0.05 to *p* < 0.001).

#### 3.2.4. Serum Biochemistry

##### 3.2.4.1. Serum Electrolytes

Table 3 summarizes the dose‐dependent alterations in serum electrolyte parameters in female Wistar rats following 28‐day oral administration of *P. fluorescens* DS17R chloroform extract. Regarding sodium levels, a clear and progressive dose‐dependent decrease was observed across all treated groups. Compared with the control (176.40  mmol/L), sodium concentrations declined significantly from 57.5 mg/kg bw onward (*p* < 0.05), reaching marked hyponatremia at 230 and 460 mg/kg bw (*p* < 0.01). This consistent downward trend suggests impaired sodium homeostasis, potentially reflecting renal tubular dysfunction, altered aldosterone‐mediated regulation, or fluid balance disturbances. Similarly, potassium levels showed a significant reduction across doses. Although the decrease was moderate at 57.5 and 115 mg/kg bw (*p* < 0.05), it became more pronounced at 230 and 460 mg/kg bw (*p* < 0.01 to *p* < 0.001), indicating hypokalemia. In parallel, chloride concentrations also decreased significantly in all treated groups, with stronger statistical significance at doses ≥ 115 mg/kg bw. However, when renal function was assessed through serum creatinine levels, no statistically significant differences were observed across groups. Creatinine values remained stable, indicating that glomerular filtration rate (GFR) was not overtly compromised. Turning to divalent ions, calcium levels exhibited a significant and dose‐dependent increase, with strong statistical significance at 230 and 460 mg/kg bw (*p* < 0.001). Conversely, phosphate levels decreased progressively and significantly across all treated groups, reaching high statistical significance at higher doses (*p* < 0.001).

**TABLE 3:** Dose‐dependent electrolyte imbalances in female Wistar rats following 28‐day oral administration of *P. fluorescens* DS17R chloroform extract.

	Control	Doses of extracts (mg/kg bw)			
		57.5	115	230	460
Sodium (mmol/L)	176.40 ± 1.66	161.33 ± 0.73^∗^	157.20 ± 2.45^∗^	134.66 ± 3.66^∗∗^	131.63 ± 1.33^∗∗^
Potassium (mmol/L)	8.29 ± 1.55	8.01 ± 0.10^∗^	7.98 ± 1.50^∗^	6.01 ± 0.32^∗∗^	6.33 ± 1.25^∗∗∗^
Chlore (mmol/L)	96.11 ± 1.32	90.40 ± 0.41^∗^	83.55 ± 4.30^∗∗^	85.29 ± 1.52^∗∗^	81.72 ± 0.24^∗∗^
Serum creatinine (μmol/L)	5.42 ± 0.02	5.38 ± 0.04 ^ns^	5.47 ± 0.52^ns^	5.37 ± 0.01^ns^	5.41 ± 0.33^ns^
Calcium (mmol/L)	2.52 ± 0.008	2.78 ± 0.002^∗∗^	2.62 ± 0.003^∗^	2.91 ± 0.007^∗∗∗^	2.94 ± 0.005^∗∗∗^
Phosphate (mmol/L)	1.98 ± 0.01	1.73 ± 0.01^∗^	1.62 ± 0.021^∗∗^	1.41 ± 0.50^∗∗∗^	1.39 ± 0.16^∗∗∗^

*Note:* Values represent mean ± SEM (*n* = 5). Statistical significance versus control: ^∗^ *p* < 0.05, ^∗∗^ *p* < 0.01, ^∗∗∗^ *p* < 0.001.

^ns^Not significant (one‐way ANOVA with Tukey’s post hoc test).

##### 3.2.4.2. Renal Urinary Biomarkers

Table 4 presents the effects of 28‐day oral administration of *P. fluorescens* DS17R chloroform extract on renal function markers and urinary biochemical parameters in female Wistar rats. Regarding serum creatinine, values remained statistically unchanged across most treated groups compared with the control, except for a slight but significant increase at 460 mg/kg bw (*p* < 0.05). Since creatinine is a classical indicator of GFR, the overall stability of this parameter suggests preserved glomerular filtration capacity throughout the exposure period. In contrast, urea concentrations exhibited a significant and marked dose‐dependent decrease from 57.5 mg/kg bw onward (*p* < 0.05 to *p* < 0.01). Similarly, uric acid levels showed no statistically significant variation across all treated groups. However, a markedly different pattern emerged when examining total protein levels. Serum protein concentrations decreased significantly and consistently across all treated doses (*p* < 0.05), with the most pronounced reduction observed at 460 mg/kg bw.

**TABLE 4:** Renal function and urinary biomarker alterations in female Wistar rats following 28‐day oral administration of *P. fluorescens* DS17R chloroform extract.

	Doses of extracts (mg/kg bw)				
	Control	57.5	115	230	460
Creatinine (μmol/L)	4.12 ± 0.02	4.15 ± 0.09^ns^	4.08 ± 0.65^ns^	4.11 ± 0.02^ns^	4.28 ± 0.20^∗^
Urea (mmol/L)	7.41 ± 0.57	5.99 ± 0.32^∗^	3.61 ± 0.12^∗∗^	2.95 ± 0.48^∗∗^	3.00 ± 0.1^∗∗^
Uric acid (mmol/L)	0.03 ± 0.001	0.04 ± 0.00^ns^	0.05 ± 0.00^ns^	0.04 ± 0.001^ns^	0.06 ± 0.002^ns^
Protein (g/L)	40.20 ± 0.65	17.58 ± 0.32^∗^	18.66 ± 0.50^∗^	16.99 ± 1.37^∗^	10.76 ± 0.94^∗^

*Note:* Values represent mean ± SEM (*n* = 5). Statistical significance versus control: ^∗^ *p* < 0.05, ^∗∗^ *p* < 0.01.

^ns^Not significant (one‐way ANOVA with Tukey’s post hoc test).

##### 3.2.4.3. Hepatic Biochemical Parameters

Table 5 presents hepatic biochemical and protein metabolism parameters in female Wistar rats following 28‐day oral exposure to *P. fluorescens* DS17R chloroform extract. Alanineaminotransferase (ALT), a sensitive marker of hepatocellular cytolysis, did not differ significantly across treated groups compared with control. Similarly, aspartate aminotransferase (AST) remained statistically unchanged at lower and intermediate doses; however, a mild but significant increase was observed at 460 mg/kg bw (*p* < 0.05) bw. In contrast to transaminases, alkaline phosphatase (ALP) exhibited a marked and dose‐dependent increase, reaching statistical significance from 115 mg/kg bw onward and becoming highly significant at 460 mg/kg bw (*p* < 0.01). Indeed, bilirubin levels remained stable across all groups, indicating preserved bilirubin conjugation and excretory capacity. Turning to hepatic synthetic function, albumin concentrations increased significantly at 230 and 460 mg/kg bw (*p* < 0.05 to *p* < 0.01). However, total protein levels showed a significant decrease at doses ≥ 115 mg/kg bw (*p* < 0.05). This apparent paradox, which elevated albumin but reduced total protein, implies a shift in plasma protein fractions. Importantly, coagulation parameters including prothrombin ratio (PTR) and plasma fibrinogen (PTF) remained statistically unchanged across all groups.

**TABLE 5:** Hepatic function and protein metabolism parameters in female Wistar rats following 28‐day oral administration of *P. fluorescens* DS17R chloroform extract.

Biochemical parameters	Doses of extracts (mg/kg bw)				
	Control	57.5	115	230	460
ALT	143.21 ± 2.45	146.34 ± 1.23^ns^	140.99 ± 4.32^ns^	145.89 ± 5.55^ns^	139.95 ± 8.02^ns^
AST	135.12 ± 0.00	137.28 ± 4.93^ns^	134.09 ± 1.23^ns^	139.82 ± 0.00^ns^	142.53 ± 6.17^∗^
ALP	46.32 ± 2.65	49.05 ± 1.91^ns^	81.75 ± 0.00^∗^	79.03 ± 6.56^∗^	120.72 ± 3.85^∗∗^
Bilirubin	0.98 ± 0.03	0.96 ± 0.25^ns^	0.94 ± 0.01^ns^	0.99 ± 0.01^ns^	1.01 ± 0.04^ns^
Albumin	4.51 ± 0.19	4.48 ± 0.66^ns^	4.55 ± 0.22^ns^	5.11 ± 0.52^∗^	5.25 ± 0.87^∗∗^
Protein	110.32 ± 0.90	107.70 ± 0.00^ns^	97.88 ± 0.33^∗^	95.12 ± 0.84^∗^	97.16 ± 1.24^∗^
PTR	22.73 ± 6.87	20.46 ± 5.82^ns^	23.63 ± 0.42^ns^	23.81 ± 5.31^ns^	27.40 ± 4.58^ns^
PTF	57.31 ± 6.95	53.19 ± 3.46^ns^	55.76 ± 4.62^ns^	60.54 ± 6.20^ns^	62.94 ± 4.28^ns^

*Note:* Values represent mean ± SD (*n* = 5). Statistical significance versus control: ^∗^ *p* < 0.05, ^∗∗^ *p* < 0.01. ALT: alanine aminotransferase, AST: aspartate aminotransferase, ALP: alkaline phosphatase, PTR: prothrombin ratio, PTF: plasma fibrinogen.

^ns^Not significant (Newman–Keuls test).

##### 3.2.4.4. Lipid Profile

Table 6 presents the effects of 28‐day oral administration of *P. fluorescens* DS17R chloroform extract on lipid profile parameters in female Wistar rats. Total cholesterol levels remained statistically unchanged at 57.5, 115, and 230 mg/kg bw compared with control. However, at 460 mg/kg bw, a modest but significant reduction was observed (*p* < 0.05). Although the decrease is quantitatively moderate, its statistical significance at the highest dose suggests that prolonged exposure may influence systemic cholesterol homeostasis. In contrast, triglyceride concentrations exhibited a clear and significant dose‐dependent increase from 57.5 mg/kg bw onward (*p* < 0.05 to *p* < 0.01). The elevation became more pronounced at 230 and 460 mg/kg bw. Simultaneously, HDL cholesterol levels increased markedly and dose‐dependently, reaching high statistical significance at higher doses (*p* < 0.001 at 460 mg/kg bw). Conversely, LDL cholesterol concentrations decreased dramatically and progressively across all treated groups, with strong statistical significance from 115 mg/kg bw onward (*p* < 0.01 to *p* < 0.001).

**TABLE 6:** Comprehensive lipid profile modulations in female Wistar rats following 28‐day oral administration of *P. fluorescens* DS17R chloroform extract.

Lipid profile	Control	Doses of extracts (mg/kg bw)			
		57.5	115	230	460
Total cholesterol (mmol/L)	0.98 ± 0.01	0.95 ± 0.01 ^ns^	0.96 ± 0.02^ns^	0.93 ± 0.01^ns^	0.90 ± 0.02^∗^
Triglycerides (mmol/L)	0.55 ± 0.009	0.66 ± 0.002^∗^	0.64 ± 0.009^∗^	0.84 ± 0.01^∗∗^	0.82 ± 0.03^∗∗^
HDL cholesterol (mmol/L)	0.14 ± 0.001	0.27 ± 0.003^∗^	0.64 ± 0.001^∗∗^	0.60 ± 0.00^∗∗^	0.70 ± 0.002^∗∗∗^
LDL cholesterol (mmol/L)	0.73 ± 0.005	0.54 ± 0.02^∗^	0.20 ± 0.001^∗∗^	0.17 ± 0.03^∗∗^	0.04 ± 0.00^∗∗∗^

*Note:* Values represent mean ± SEM (*n* = 5). Statistical significance versus control: ^∗^ *p* < 0.05, ^∗∗^ *p* < 0.01, ^∗∗∗^ *p* < 0.001.

Abbreviations: HDL, high‐density lipoprotein; LDL, low‐density lipoprotein.

^ns^Not significant (one‐way ANOVA with Tukey’s post hoc test).

#### 3.2.5. Histopathological Examination

**FIGURE 3:**
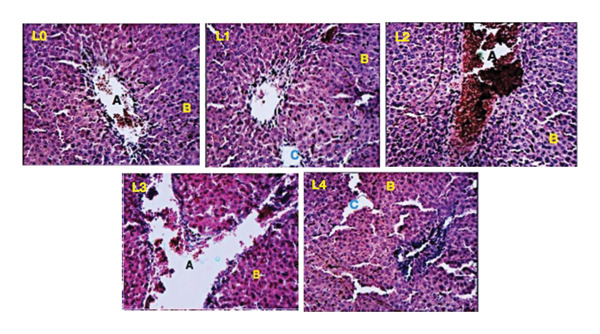
Liver (L) sections of female rats showing effects of oral administration of *P. fluorescens* extract over 28 days; L0: control group; L1: 57.5 mg/kg bw; L2: 115 mg/kg bw; L3: 230 mg/kg bw; L4: 460 mg/kg bw. Indicators: (A) branch of the hepatic portal vein; (B) hepatocytes; (C) leukocyte infiltration (inflammation).

**FIGURE 4:**
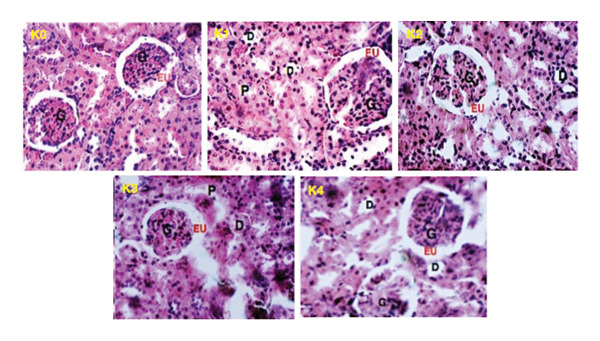
Kidney (K) sections of female rats showing effects of oral administration of *P. fluorescens* extract over 28 days; K0: control group; K1: 57.5 mg/kg bw; K2: 115 mg/kg bw; K3: 230 mg/kg bw; K4: 460 mg/kg bw. Indicators: (G) glomerulus; (EU) urinary space; (P) proximal convoluted tubule; (D) Distal convoluted tubule.

Histopathological analysis of liver tissues (Figure 3) from treated animals revealed dose‐dependent alterations. While the control group (L0) displayed normal hepatic architecture, treated groups exhibited sinusoidal dilation (L1, L2, L3) and leukocyte infiltration (L3, L4), suggesting inflammatory or oxidative stress. The portal vein structure remained intact in all groups. In renal tissues (Figure 4), the overall architecture of glomeruli and tubules was preserved at lower doses. However, at higher doses of K2, K3, and K4 (115, 230, and 460 mg/kg bw), localized vascular congestion and tubular lumen enlargement were observed, indicating mild renal stress.

## 4. Discussion

The present study provides a comprehensive acute and subacute toxicological characterization of the chloroform extract of *Pseudomonas fluorescens* DS17R following oral administration in female Wistar rats. Acute toxicity testing classified the extract as practically nontoxic, reflecting a favorable safety profile while revealing dose‐ and sex‐dependent sensitivities [32]. The findings demonstrate that repeated exposure induces a complex pattern of dose‐ and time‐dependent physiological modulation involving metabolic, hematological, renal, hepatic, and immunological pathways. Importantly, the data reveal adaptive responses at lower doses and emerging functional disturbances at higher doses, underscoring the necessity of subacute evaluation in microbial‐derived bioactive preparations [37]. The evolution of body weight and feed intake provides the first integrative indicator of systemic tolerance. During the second and third weeks, a nonmonotonic pattern was observed, with increased body weight at 57.5 mg/kg bw suggesting a possible hormetic response. Hormesis, characterized by low‐dose stimulation and high‐dose inhibition, has been widely described in toxicology and reflects adaptive metabolic activation at subthreshold exposure levels [37]. However, by week four, a marked reduction in relative body weight was evident at doses ≥ 115 mg/kg bw, particularly pronounced at 115 mg/kg bw, indicating cumulative or delayed toxicity. This reduction coincided temporally with altered feed intake patterns. Although early weeks showed fluctuating consumption without clear monotonicity, week two revealed significant suppression at 230 mg/kg bw, and by week four, persistent reductions were evident at intermediate doses. The partial recovery of feed intake at 460 mg/kg bw during later stages may reflect metabolic adaptation or compensatory behavioral mechanisms rather than the absence of toxicity. In repeated‐dose toxicology, sustained body weight suppression exceeding 10% is considered biologically relevant and indicative of systemic stress [32]. Therefore, the observed late‐phase weight reduction supports the presence of cumulative metabolic burden. Organ weight analysis further clarifies target organ sensitivity. The liver demonstrated a clear and statistically robust dose‐dependent enlargement across all treated groups, reaching high significance at 230 and 460 mg/kg bw. Hepatomegaly is classically interpreted as either hepatocellular hypertrophy secondary to enzyme induction or inflammatory infiltration [38]. The concomitant increase in ALP and mild elevation of AST at the highest dose strongly support hepatic enzymatic induction and mitochondrial stress rather than overt necrosis, particularly in light of unchanged ALT and bilirubin levels [39, 40]. ALT is more liver‐specific and correlates with cytoplasmic membrane damage, whereas AST elevation may reflect mitochondrial perturbation [40]. Histologically, sinusoidal dilation and leukocyte infiltration corroborate a mild inflammatory response without architectural collapse. The preserved portal structures further argue against advanced hepatotoxicity. Collectively, these findings suggest adaptive metabolic activation accompanied by low‐grade inflammatory stress [41]. In parallel, splenic enlargement exhibited a clear dose‐dependent increase, becoming highly significant at 460 mg/kg bw. Splenomegaly frequently reflects immune activation or altered erythrocyte turnover [42]. This observation aligns with the hematological findings, where erythrocyte parameters showed complex modulation. Hb levels increased significantly from 115 mg/kg bw onward, whereas total RBC counts, PCV, and MCV decreased dose‐dependently. The simultaneous reduction in MCV and increase in MCHC, coupled with elevated RDW, indicates microcytosis with anisocytosis. Such a profile suggests altered erythropoiesis or increased erythrocyte turnover. Oxidative stress‐mediated membrane damage is known to reduce erythrocyte lifespan and stimulate compensatory erythropoietic responses [43]. The increase in PLT (thrombocytosis) may similarly reflect bone marrow stimulation or inflammatory signaling. Therefore, splenic enlargement likely represents both immune and hematopoietic activation. Conversely, leukocyte counts declined significantly across treated groups, with dose‐dependent lymphopenia at higher doses. Lymphocyte reduction, in the absence of neutrophil changes, suggests selective immunomodulation rather than generalized myelosuppression. This pattern of selective lymphopenia without neutropenia has been described in response to bacterial metabolites that induce apoptosis in lymphocyte populations while sparing myeloid lineages [44]. The concurrent monocytosis may represent compensatory inflammatory recruitment, consistent with immune activation patterns observed following exposure to immunomodulatory xenobiotics [45, 46]. Phenazine‐type metabolites produced by *Pseudomonas* species are known to generate reactive oxygen species via redox cycling, leading to lymphocyte apoptosis [47, 48]. The observed monocytosis may represent compensatory inflammatory recruitment. Importantly, eosinophils remained stable, excluding allergic‐type immune activation. The observed pattern of microcytosis, anisocytosis, and lymphopenia is consistent with oxidative stress‐mediated erythrocyte damage and lymphocyte apoptosis, mechanisms well‐documented for phenazine‐producing bacteria [21]. Thus, hematological findings point toward oxidative and inflammatory modulation rather than cytotoxic marrow suppression [49]. Renal function assessment revealed a nuanced pattern. Serum creatinine remained largely stable except for a slight increase at 460 mg/kg bw, indicating preserved glomerular filtration until high‐dose exposure [50]. However, significant and progressive hyponatremia, hypokalemia, and hypochloremia were observed, accompanied by hypercalcemia and hypophosphatemia. Electrolyte imbalance in the absence of marked creatinine elevation suggests tubular dysfunction rather than primary glomerular failure [50–52]. Recent advances in nephrotoxicology have elucidated the molecular pathways by which xenobiotics induce tubular dysfunction without immediately compromising glomerular filtration. Oxidative stress impairs energy‐dependent tubular transport mechanisms, particularly sodium‐potassium ATPase activity, leading to urinary electrolyte wasting. Furthermore, mitochondrial dysfunction in proximal tubular cells can disrupt phosphate reabsorption, explaining the observed hypophosphatemia. The histopathological findings of vascular congestion and tubular dilation at higher doses provide morphological correlates to these functional disturbances. Tubular handling of sodium and potassium is highly energy‐dependent and vulnerable to oxidative stress [51, 52]. The reduction in serum total protein and urea further supports altered renal or hepatic metabolic handling. Histopathological findings of vascular congestion and tubular dilation at higher doses provide morphological confirmation of mild renal stress. Since electrolyte disturbances can precede structural nephropathy, these changes likely represent early functional toxicity. The hepatic synthetic profile provides additional insight. Albumin levels increased at higher doses, while total protein decreased. This divergence indicates redistribution among plasma protein fractions rather than global hepatic insufficiency. Moreover, coagulation parameters (PTR and fibrinogen) remained stable, confirming preserved hepatic synthetic capacity [39]. Therefore, the biochemical pattern reflects selective enzymatic adaptation rather than hepatic failure. Perhaps the most striking systemic effect was the remodeling of lipid metabolism. Triglycerides increased significantly and dose‐dependently, while LDL cholesterol decreased dramatically and HDL cholesterol rose markedly. Total cholesterol declined modestly only at the highest dose. This lipid dissociation suggests selective modulation of hepatic lipid transport and lipoprotein metabolism. Hypertriglyceridemia may result from enhanced de novo lipogenesis or reduced peripheral clearance, potentially mediated by xenobiotic‐sensitive transcription factors such as SREBP‐1c and PPARα [50, 53]. PPAR‐α activation, in particular, has been shown to increase hepatic fatty acid oxidation while simultaneously modulating lipoprotein metabolism, leading to increased HDL synthesis and enhanced LDL receptor expression [54]. The pattern observed in this study increased HDL, decreased LDL, and elevated triglycerides is consistent with partial PPAR‐α agonism, although the concurrent hypertriglyceridemia suggests additional effects on VLDL secretion or clearance [55]. Meanwhile, elevated HDL and reduced LDL may reflect altered reverse cholesterol transport dynamics [56]. Although these changes appear cardiometabolically favorable in human clinical contexts, rodent lipid physiology differs substantially, limiting translational interpretation [56]. In toxicological terms, the triglyceride increase is more relevant, as persistent hypertriglyceridemia can signal hepatic metabolic strain [53, 57]. Importantly, when all findings are integrated, a coherent mechanistic hypothesis emerges. The combination of hepatic hypertrophy, mild AST elevation, lymphopenia, splenic enlargement, triglyceride increase, and electrolyte imbalance converges toward redox‐mediated stress as a central pathway [47, 48]. Phenazine‐like secondary metabolites capable of generating reactive oxygen species provide a biologically plausible unifying mechanism. Reactive oxygen species can disrupt mitochondrial respiration, alter lipid synthesis pathways, induce immune cell apoptosis, and impair renal tubular transport [47, 48, 58]. The histopathological evidence of inflammatory infiltration in the liver and vascular congestion in the kidney supports this integrated oxidative‐inflammation model. Nevertheless, it is critical to distinguish adaptive from adverse responses. Although multiple parameters reached statistical significance, most remained within physiological ranges and did not culminate in irreversible structural damage [59]. According to contemporary toxicological risk frameworks, adversity requires persistent functional impairment or progressive pathology [59]. Within the 28‐day exposure window, the extract induced multisystem biological activity but did not produce overt organ failure. However, the highest dose (460 mg/kg bw) approached a threshold where adaptive mechanisms may transition toward toxicity. Several limitations warrant acknowledgment, and longer exposure durations could be necessary to determine reversibility or progression. Repeated oral exposure to *P. fluorescens* DS17R chloroform extract induces time‐ and dose‐dependent systemic physiological modulation characterized by transient hormetic effects at low doses, cumulative body weight suppression at intermediate doses, hepatic enzymatic induction with hepatomegaly, splenic and hematopoietic activation, selective lymphopenia, renal tubular electrolyte imbalance, and significant lipid remodeling marked by hypertriglyceridemia and HDL elevation. These findings collectively support a model of adaptive metabolic and redox‐mediated stress rather than overt organ toxicity within 28 days, while highlighting the importance of extended‐duration studies to define long‐term safety margins.

## 5. Conclusion

In this study, *Pseudomonas fluorescens* DS17R demonstrates a promising profile as a microbial biocontrol agent, exhibiting practically nontoxic acute oral effects, while its subacute toxicity profile reveals complex, dose‐ and time‐dependent physiological adaptations. Repeated 28‐day oral administration of the chloroform extract at 57.5, 115, 230, and 460 mg/kg body weight (bw) induced multifaceted systemic responses in Wistar rats. Low‐dose exposure (57.5 mg/kg bw) elicited mild weight gain indicative of hormetic stimulation, whereas intermediate and high doses (115–460 mg/kg bw) led to cumulative body weight suppression, transient reductions in feed intake, and hepatomegaly with elevated ALP and mild AST elevation. Histopathological evaluation confirmed sinusoidal dilation and leukocyte infiltration without overt structural liver damage. Dose‐dependent splenic enlargement, lymphopenia at 230–460 mg/kg bw, microcytosis with anisocytosis, and thrombocytosis reflected hematopoietic and immune adaptation. Renal assessments revealed hyponatremia, hypokalemia, hypochloremia, hypercalcemia, and phosphate depletion, accompanied by mild tubular alterations, while glomerular filtration remained preserved. Lipid metabolism was selectively modulated, with increased triglycerides and HDL and decreased LDL at doses ≥ 115 mg/kg bw. Collectively, these findings indicate adaptive, multisystem metabolic and redox‐mediated responses rather than overt organ toxicity, establishing a rigorous toxicological profile supportive of the safe development of *P. fluorescens* DS17R for agricultural application. Further long‐term studies are warranted to delineate the reversibility of observed effects and refine safety margins for chronic exposure scenarios.

## Author Contributions

Samuel Arsène Ntyam Mendo carried out the study; Samuel Arsène Ntyam Mendo, Laure Brigitte Kouitcheu Mabeku, Séverin Tchameni Nguemezi designed the experiments, Modeste Lambert Sameza and Samuel Arsène Ntyam Mendo wrote the manuscript; Rosalie Anne Ngono Ngane and Laure Brigitte Kouitcheu Mabeku supervised the work.

## Funding

The authors have nothing to report.

## Disclosure

All authors read and approved the final manuscript.

## Ethics Statement

The authors have nothing to report.

## Conflicts of Interest

The authors declare no conflicts of interest.

## Data Availability

The data that support the findings of this study are available from the corresponding author upon reasonable request.
